# Correction to: Secukinumab: A Review in Ankylosing Spondylitis

**DOI:** 10.1007/s40265-019-01089-x

**Published:** 2019-03-11

**Authors:** Hannah A. Blair

**Affiliations:** Springer, Private Bag 65901, Mairangi Bay, Auckland, 0754 New Zealand

## Correction to: Drugs 10.1007/s40265-019-01075-3

The article Secukinumab: A Review in Ankylosing Spondylitis, written by Hannah A. Blair, was originally published Online First without open access. After online publication Novartis Pharma AG requested that the article be Open Choice to make the article an open access publication. Post-publication open access was funded by Novartis Pharma AG. The article is forthwith distributed under the terms of the Creative Commons Attribution-NonCommercial 4.0 International License (http://creativecommons.org/licenses/by-nc/4.0/), which permits any noncommercial use, duplication, adaptation, distribution and reproduction in any medium or format, as long as you give appropriate credit to the original author(s) and the source, provide a link to the Creative Commons license and indicate if changes were made.

The original article has been update.

